# Acute and lagged fitness consequences for a sagebrush obligate in a post mega‐wildfire landscape

**DOI:** 10.1002/ece3.8488

**Published:** 2021-12-24

**Authors:** Christopher R. Anthony, Lee J. Foster, Christian A. Hagen, Katie M. Dugger

**Affiliations:** ^1^ U.S. Geological Survey Oregon Cooperative Fish and Wildlife Research Unit Department of Fisheries, Wildlife, and Conservation Sciences Oregon State University Corvallis Oregon USA; ^2^ Oregon Department of Fish and Wildlife Hines Oregon USA; ^3^ Department of Fisheries, Wildlife, and Conservation Sciences Oregon State University Corvallis Oregon USA

**Keywords:** *Centrocercus urophasianus*, demographics, fire, life history, sagebrush, survival

## Abstract

Species responses to disturbance influence their extinction risks. Greater sage‐grouse (*Centrocercus urophasianus*) are bioindicators of sagebrush ecosystem health and the loss of sagebrush (*Artemisia* spp.) due to wildfire, can cause long‐term declines in sage‐grouse populations and other sagebrush obligate species. We examined the demographic response of a greater sage‐grouse population following a mega‐wildfire using stochastic age‐structured female‐based matrix models over 6 years (2013–2018). Notably, chick survival (range = 0.18–0.38) and female survival (yearling range: 0.20–0.68; adult range: 0.27–0.75) were low compared to values reported for greater sage‐grouse in other parts of their distribution. Greater sage‐grouse displayed variation in demographic tactics after the fire; however, adult female survival explained most of the variation in *λ* during each year, which reflected a declining population in 3 of 6 years with more uncertainty observed in 2015 when populations may have been increasing, and 2017 and 2018, when populations may have been declining. The continued annual population decline observed since 2016 suggested there were additional strong environmental impacts that may have been compounded by the fire effects, prolonging recovery of greater sage‐grouse. Our results support others that reported negative effects to greater sage‐grouse demographics from broad‐scale fire and provide a baseline for understanding how this species responds to loss of sagebrush cover based on their life history strategy.

## Graphical abstract

Chick survival and female survival were low for Greater sage‐grouse a megafire, compared to values reported throughout their range. Greater sage‐grouse exhibited variation in demographic tactics; however, adult female survival explained most of the variation in the finite rate of population change, which reflected a declining population in 3 of 6 years following the fire.

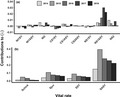

## 1 INTRODUCTION

Accurate estimation and prediction of demographic vital rates that regulate and limit populations is fundamental for wildlife conservation (Williams et al., 2002). Considering vital rates within the “slow‐fast continuum” is a useful framework for guiding management of wildlife populations with varying life‐history strategies (Jones, 1976; Pianka, 1970; Salguero‐Gómez et al., 2016; Stearns, 1976). Species on the slow end of the continuum can afford to delay reproduction, or reproduce at relatively low rates when they do breed because they are long‐lived, with many opportunities to produce offspring (k‐selected; Promislow & Harvey, 1990; Saether, 1988). Conversely, species on the fast end of the spectrum have shorter generation times and need to produce offspring early in life to pass along their genes to the next generation (r‐selected; Promislow & Harvey, 1990; Saether, 1988). Most gallinaceous bird species have low adult survival and high reproductive rates, which would place gallinaceous birds on the fast end of the continuum (Gaillard et al., 1989; Johnsgard, 1983). However, greater sage‐grouse (*Centrocercus urophasianus*; hereafter, sage‐grouse) are characterized as having relatively low reproductive output and high annual survival compared to other galliformes (Apa, 1998; Schroeder et al., 1999), and their life history strategy is likely somewhere in the middle of the slow‐fast life history continuum for birds in general (Koons et al., 2014).

Life history strategies evolve in response to environmental conditions and knowledge of how a species responds to disturbance can help assess their risk of extinction (Pianka, 1970; Reznick et al., 2002; Stearns, 1989). Sage‐grouse and other species associated with sagebrush (*Artemisia* spp.) have co‐evolved in sagebrush communities where wildfire is dynamic, albeit somewhat predictable at broad spatial and temporal scales (Baker, 2006; Miller et al., 2011). However, during the last 35 years, the frequency of mega‐wildfires (>40,000 ha) have increased in the Great Basin and this shift has been linked to declines in sage‐grouse populations (Brooks et al., 2015; Coates et al., 2016). Because sage‐grouse require sagebrush during all phases of their life cycle and share similar habitat requirements with other sagebrush‐obligate species, they are considered a bioindicator of sagebrush ecosystem health (Copeland et al., 2014; Hanser & Knick, 2011). The effects of fire on sage‐grouse populations are strongly linked to the reduction of sagebrush cover within their seasonal ranges that occurs after fire (Coates et al., 2016). The response of sage‐grouse to the loss of sagebrush is overwhelmingly negative across their range as disturbances that remove or render sagebrush unusable have demographic consequences (Aldridge & Boyce, 2007; Dzialak et al., 2011; Foster et al., 2019; Kirol et al., 2015; LeBeau et al., 2014). Wildfire that kills sagebrush has negative consequences on lek attendance (Coates et al., 2015, 2016; Dudley et al., 2021; Hess & Beck, 2012; Steenvoorden et al., 2019) and key vital rates like nest survival (NS) (Anthony et al., 2021; Foster et al., 2019; Lockyer et al., 2015) and adult female survival (Foster et al., 2019). Even smaller sized prescribed fires (<6000 ha) that remove sagebrush have been associated with declines in sage‐grouse populations (Connelly et al., 2000; Fischer, 1994). While the negative effects of wildfire on some important sage‐grouse vital rates are becoming apparent, the influence of these outcomes to long‐term patterns in population change is still poorly understood (Coates et al., 2016). Thus, there is a need to identify the life‐history stages that are most important following wildfire, so that restoration can be appropriately targeted to have the greatest impact on population growth.

Vital rates most important for driving sage‐grouse population change (*λ*) can vary among populations (e.g., Walker & Naugle, 2011 vs. Dahlgren et al., 2016). For example, several studies have reported *λ* to be most sensitive to changes in adult female survival, juvenile (fledging to 1‐year old) survival, and chick survival (Dahlgren et al., 2016; Johnson & Braun, 1999; Olsen et al., 2021; Taylor et al., 2012). However, nest success explained most of the variation in *λ* across multiple populations representing the current sage‐grouse distribution, in part due to the high temporal variation in this vital rate (Taylor et al., 2012). Generally, vital rates that are important drivers of *λ* have low temporal variation, but in some instances the opposite pattern has been observed (i.e., those with a large effect on *λ* can have high temporal variation; Dahlgren et al., 2016). Still, adult survival in sage‐grouse populations might be expected to have a greater effect on *λ* compared to other grouse species because they occur on the slower end of the life history continuum compared to most galliformes (Koons et al., 2014). The exception might be populations that are located in fragmented landscapes or those which are less stable, as they might be more sensitive to vital rates associated with fecundity (Fefferman & Reed, 2006).

Given the paucity of information linking interactions among life‐history strategies and wildfire, there is a need to better understand how these mechanisms influence *λ* in sage‐grouse populations affected by mega‐wildfire. Our objective was to quantify the demographic and population response of sage‐grouse over 6‐years (2013–2018) following a mega‐wildfire. Furthermore, we calculated the proportional contribution of each sage‐grouse vital rate to the overall variation in *λ* and assessed which of these vital rates had the greatest influence on *λ*.

## 2 METHODS

### 2.1 Study area

Our study was conducted in the Trout Creek Mountains in southeast Oregon and northwest Nevada, within the Great Basin. Annual precipitation and elevation ranged from 200 to 600 mm and 1372 m to 2593 m, respectively. The study area was delineated by the Holloway fire perimeter that burned 186,972 ha during the summer of 2012. Within the fire perimeter, 75% of the land area burned, leaving 25% unburned vegetation of various size patches (largest unburned patch ~4000 ha; Foster et al., 2018). Unburned sagebrush communities were dominated by Wyoming big sagebrush (*Artemisia tridentata wyomingensis*) and low sagebrush (*A*. *arbuscula*) at lower elevations and a mosaic of a mountain big sagebrush (*A*. *t*. *vaseyana*) and low sagebrush at higher elevations. Burned sagebrush communities varied in composition and structure and were representative of much of the Great Basin following wildfire (Miller et al., 2013). Generally, Wyoming big sagebrush communities with warm‐dry soils exhibited low species diversity, low sagebrush cover, and high cheatgrass (*Bromus tectorum*) cover (Anthony et al., 2020). Whereas, mountain sagebrush communities with cool‐moist soils had high species diversity, moderate sagebrush cover, and low cheatgrass cover (Anthony et al., 2020).

### 2.2 Capture and vital rate monitoring

We captured female sage‐grouse within or near (≤2 km) the Holloway fire perimeter during 2013–2018 using established spotlighting methods (Wakkinen et al., 1992). We used wing characteristics to classify captured individuals into age categories (Braun & Schroeder, 2015) and attached 22‐g or 30‐g ARGOS/GPS Solar PTTs (PTT‐100, Microwave Telemetry Inc.) using a rump‐mount technique (Rappole & Tipton, 1991) to female yearlings (~1 year old) and adults (≥2 years old). Capture and handling of all individuals were conducted under protocols approved by the Institutional Animal Care and Use Committee at Oregon State University.

For most of the females, we collected 5 locations per day: 2 during the morning, 2 during the afternoon/evening, and one at night during the nesting season (1 April to 31 May). We downloaded locations every 1–2 days during the nesting season and following the observation of 3 stationary locations within 18 hours, confirmed an active nest with field observations within a few days of the GPS download. We revisited nests after they were terminated and considered a nest “successful” if ≥1 egg displayed a distinct egg cap and intact egg membrane, signifying a hatched egg, and “unsuccessful” if these criteria were not met.

We conducted brood counts every 10 days for 5 weeks (~54 days’ post‐hatch), beginning 2‐weeks post‐hatch, using either the single or double observer method to actively flush birds (Dahlgren et al., 2010). We counted marked females and their chicks by locating females with UHF handheld receivers and Yagi antennas and walking a 50 m grid at 2–4 m transects around the location where the marked female was flushed. We conducted another brood count within 1–3 days if a marked female was not detected or behaved as if she did not have chicks (i.e., flushed and flew long distances, and no feigning injury). If no chicks were observed with the marked female after two counts, then we considered the brood “failed.”

We used GPS location data to examine movement patterns by female sage‐grouse and identify potential mortalities. We considered potential mortality events to have occurred when GPS locations remained stationary for >18 h for a female. We confirmed mortality events in the field using last known GPS locations and a UHF receiver and antenna. We considered events “mortalities” if the GPS‐PTT was located and there were ≥1 conclusive signs of mortality such as feathers, bone fragments, or damage to the transmitter. We assigned the date of the mortality as the last known movement by the female.

### 2.3 Vital rate estimates

We estimated age‐specific annual nest incubation initiation rates (NI) for first nests as the number of females that began incubating, divided by the number of females that were monitored and alive through the first nesting period (i.e., last individual to begin incubation for first nests). Sample sizes for re‐nests by age class were small each year, therefore, we pooled yearlings and adults and estimated annual nest initiation rate as the proportion of all females that were monitored through the second nesting period (i.e., last individual to begin incubation for re‐nests) and began incubating a second nest, given the first nest failed. Therefore, we had a single annual re‐nesting rate that was used to reflect re‐nesting of both age classes. We estimated standard errors for nest initiation rate based on the variance of the sample proportion (*σ* ^2^ = *pq*/*n*), where *p* = number of females that nested, *q* = 1–*p*, and *n* = number of females that were monitored through the entire nesting period.

We estimated age‐specific mean annual clutch size (CS) for first nests as the total number of eggs counted after the nest was terminated or during nesting if the female was flushed during monitoring, divided by the number of total nests for yearlings and adults. For CS estimates of re‐nests, we used results from a meta‐analysis of CSs across the range of sage‐grouse (Taylor et al., 2012) that reported the mean difference between mean CS for first nests and re‐nests (1.39 eggs for yearlings; 1.63 eggs for adults). Therefore, to estimate CS for re‐nests we subtracted 1.39 eggs (yearlings) and 1.63 eggs (adults) from mean annual clutch size estimates of first nests. We calculated standard deviation of CS for re‐nests by age (yearling, *n* = 8; adult, *n* = 9) following Taylor et al. (2012).

We estimated egg hatch success rate (i.e., hatchability; *H*) for all years as the total number of eggs hatched divided by the number of total eggs laid for all nests that were successful. We estimated standard errors for hatchability based on the variance of the sample proportion.

We did not have data on the survival of juveniles (54‐days post hatch to first‐breeding; ~April); therefore, we derived estimates of juvenile survival (*S*_juv_) from annual adult survival estimates. We used an adjustment rate based on Apa et al. (2017) that examined juvenile survival (0.53) relative to adult survival (0.83) for 183 individuals over 7 months in Colorado from 2005 to 2008. Therefore, we scaled adult survival from our study area to a 7‐month time period comparable to the juvenile survival period by raising annual adult survival to the 0.58th power (7/12 = 0.58). Then, we multiplied the scaled 7‐month adult survival by 0.7 (0.53/0.83). We calculated standard error for juvenile survival following Apa et al. (2017).

For nest, chick, and female survival, we used an information theoretic approach to evaluate vital rate variation relative to age, and temporal patterns (Burnham & Anderson, 2002). We developed relevant model sets and used the Akaike Information Criteria corrected for small sample size (AIC_c_) to rank models, and differences in AIC_c_ value (∆AIC_c_) between each candidate model and the top ranked model, and AIC_c_ weights (*wᵢ* = weight of evidence supporting model *i* as the best model in the model set) to evaluate model support. We categorized year as a grouping variable in each model and model averaged estimates of nest, chick, and female survival to account for model selection uncertainty and produce the most accurate estimates of age‐specific survival for each year (Burnham & Anderson, 2002).

We estimated nest survival (NS) from the onset of incubation through hatch using the known fate model in Program MARK because the exact dates of the initiation of incubation and nest fate were known from GPS location data (White & Burnham, 1999). We did not use nests where females were flushed during nest monitoring and subsequently abandoned their nests (*n *= 2). We estimated daily nest survival (DNS) for a 27‐day incubation period each year (2013–2018) and classified year as a grouping variable. We estimated DNS for first nests in separate known fate analyses for yearlings versus adults, but then pooled data from both age classes to estimate DNS for re‐nests. We developed additive and interactive models to examine the influence of temporal patterns on DNS. Temporal covariates included (1) day: daily time‐dependent variation within the 27‐day nesting period, (2) T: increasing or decreasing linear trends within the 27‐day nesting period, and (3) Julian date: start of incubation according to 3 consecutive GPS locations. We derived estimates of NS for the 27‐day incubation period by taking the product of DNS estimates over the 27‐day nesting period for each year and estimated variance using the delta method (Powell, 2007).

We estimated chick survival (*S*_chick_) using the “young survival from marked adults” model in Program MARK, which provided estimates from counting young in a family group such as marked females and their unmarked chicks (Lukacs et al., 2004). Encounter histories reflected the number of chicks that were observed on one 14‐day post‐hatch flush, four 10‐day flush counts thereafter (5 flush counts total), and the initial count based on the number of eggs that were observed in the nest bowl after the nest was terminated (Gibson et al., 2017). We used the Lukacs model for estimated apparent survival of chicks (φ) while accounting for imperfect detection (*ρ*) similar to the Cormack‐Jolly‐Seber model (Lukacs et al., 2004). We pooled data across age classes because of small sample sizes each year. We hypothesized that the probability of detecting a brood varied temporally because chicks are capable of flight at approximately 2 weeks post‐hatch and have restricted movement, thus they tend to hide in vegetation and avoid detection (Aldridge & Brigham, 2001; Schroeder, 1997; Wallestad, 1975). Furthermore, detection could vary due to sampling error within and between years due to different observers or changes in vegetation cover post‐fire. We examined additive and interactions of the following temporal covariates: (1) count interval: variation among brood counts, and (2) T: increasing or decreasing linear trends during the brood year. To reduce the overall number of models evaluated, we evaluated the best *ρ* structure first while allowing φ to vary by year (e.g., φ(year) *ρ* (year*day); φ(year) *ρ* (T); *n* = 8). Then, we included the best *p* structure in the model set to evaluate the best temporal structure on φ based on additive and interaction combinations of time covariates (*n* = 4). We derived estimates of chick survival for the brood‐rearing period by taking the product of count interval survival estimates over the 54‐day brood‐rearing period for each year.

We estimated age‐specific monthly survival using known fate models with a staggered entry design and Program MARK (Pollock et al., 1989; White & Burnham, 1999) over a 12‐month period (March–February) each year. We used the first and last day of each calendar month to construct monthly encounter histories for each marked female during the study period. We censored individuals if their fate during a monthly interval was unknown, which only occurred if the individual was not included in the sample population or the transmitter failed and locations were not available. We considered yearling sage‐grouse to transition to adult if they were alive at the beginning of March following the year they were captured and marked. We categorized 3 seasons into 4 month increments based on biological periods and habitat characteristics: breeding (April–July), fall (August–November), and winter (December–March; Connelly, Hagen, et al., 2011; Connelly, Rinkes, et al., 2011). We developed a model set that included year and age as base structures in each model to then evaluate the additional influence of temporal covariates within a year (i.e., season, month) on survival. We defined temporal covariates as: (1) season: monthly survival was grouped seasonally, and (2) month: monthly variation within the 12‐month period. We derived seasonal age‐specific estimates by multiplying model averaged monthly survival (4 months) in each season (*S*_season_ = *S*_month1_ × *S*_month2_ × *S*_month3_ × *S*_month4_) and calculated estimates of standard error using the delta method (Powell, 2007). We derived annual estimates as the product of model averaged monthly survival from all 12 months (*S*_annual_ = *S*_month1_ × *S*_month2…_ × *S*_month12_).

The use of GPS transmitters to monitor demographics and habitat can lower survival compared to individuals wearing VHF units (5%–30%; Foster et al., 2018; Severson et al., 2019). Thus, our estimates of female survival may have been biased by the GPS devices we used to monitor sage‐grouse. During previous monitoring to estimate female survival in on our study area, sage‐grouse fitted with GPS devices had on average, 5% lower survival than those wearing VHF units (Foster et al., 2018). To account for this bias, we adjusted female survival estimates and standard errors based on the absolute difference in survival between GPS devices and VHF units from Foster et al. (2018; *S*_annual_ + 0.05).

### 2.4 Population matrix model

We developed annual, 2‐age, female stochastic population matrix models for yearlings and adults using vital rates estimated for each age class (except for hatchability, 0.95—see Section 2) and year (table 4.1, fig. 4.1, Caswell, 2001). We constructed annual 2 × 2 population matrix models using the pre‐birth‐pulse format, because we began monitoring annual female survival in March of each year, and we included fecundity (*F*) and survival (*S*) for both yearlings (SY) and adults (ASY, Caswell, 2001):

$$A=\left[\begin{array}{cc} F_{\text{SY}}S_{\text{juv}} & F_{\text{ASY}}S_{\text{juv}}\\ S_{\text{SY}} & S_{\text{ASY}} \end{array}\right]$$

We developed age‐specific fecundity (*F*) estimates using the following equation:

$$F_{j}=\left[\left\{\left({\text{NI}}_{1}*{\text{CS}}_{1}*{\text{NS}}_{1}\right)+\left(\left(1‐{\text{NS}}_{1}\right)*{\text{NI}}_{2}*{\text{CS}}_{2}*{\text{NS}}_{2}\right)\right\}*H\right]*\left(0.5*S_{\text{chick}}\right)$$

where subscript *j* represents age class, subscripts 1 and 2 indicate first nests and re‐nests, respectively, and 0.5 = portion of offspring that are female (Atamian & Sedinger, 2010).

We parameterized annual matrices with bootstrapped fecundity and annual survival estimates by resampling lower‐level vital rates 10,000 times from beta distributions for probabilities (e.g., nest survival) and normal distributions for clutch size. We estimated *λ* and 95% confidence intervals (CIs) by taking 10,000 replicates from parameterized matrices (Meyer et al., 1986). We used bootstrap estimates of *λ* and 95% confidence intervals to assess whether the population was increasing (*λ* ≥ 1.0), stable (*λ* = 1.0), or decreasing (*λ* ≤ 1.0) annually. We performed all analyses in program R version 3.6.2 (R Foundation for Statistical Computing).

### 2.5 Life table response experiment (LTRE)

We conducted a life‐table response experiment (LTRE) to understand the influence of individual vital rates (e.g., *S*_chick_) on *λ* (Caswell, 1989). The LTRE is a type of retrospective analysis that is used to quantify the contribution of individual vital rates to differences in *λ* between 2 populations under different conditions or treatments (Caswell, 1989). We used time‐since‐fire to examine the contribution of vital rates on *λ* where year 2013 (*λ* ^13^) was the reference matrix and each year *j* from 2014 to 2018 (*λ^j^*) were the treatment matrices. Contributions of vital rates to *λ* reflect the differences in each vital rate (*x_i_*) between the reference matrix (*λ* ^13^) and treatment matrices (*λ^j^*), and the sensitivity of *λ* to absolute changes in that vital rate (∂*λ*/∂*x_i_*) where:

$$\lambda^{13}‐\lambda^{j}\approx \sum_{i}\left(x_{i}^{13}‐x_{i}^{j}\right)\left(\partial \lambda /\partial x_{i}\right)$$

## 3 RESULTS

### 3.1 Vital rate estimates

We captured and attached GPS‐PTTs to 75 yearlings and 90 adult female sage‐grouse from 2013 to 2018. We monitored 177 nests and 63 broods within the study area (<2 km of the fire perimeter). Mean nest incubation initiation rates for first nests were lower for yearlings (NI_1SY_ = 0.896, 95% CI: 0.892–0.900) than for adults (NI_1ASY_ = 0.957, CI: 0.956–0.957, Table 1). Mean nest incubation initiation rates for first nests (NI_1_ = 0.939, 95% CI: 0.938–0.939) were higher compared to re‐nests (NI_2_ = 0.349, 95% CI: 0.345–0.33, Table 1). Mean clutch sizes for the first nests of adults (CS_1ASY_ = 6.43, 95% CI: 6.06–6.80) and yearlings (CS_1SY_ = 6.00, 95% CI: 5.27–6.73) were similar (Table 1). Hatchability across all years was 0.950 (95% CI: 0.948–0.952, Table 1).

**TABLE 1:** Mean vital rate estimates and standard error (SE) of female greater sage‐grouse in the Trout Creek Mountains, Harney and Malheur counties, Oregon, USA, 2013–2018

Vital rate	2013		2014		2015		2016		2017		2018	
	*n*	$\bar{x}$(SE)	*n*	$\bar{x}$(SE)	*n*	$\bar{x}$(SE)	*n*	$\bar{x}$(SE)	*n*	$\bar{x}$(SE)	*n*	$\bar{x}$(SE)
NI_1SY_	6	1.00 (0.00)	9	1.00 (0.00)	11	0.82 (0.01)	3	1.00 (0.00)	10	0.90 (0.01)	9	0.78 (0.02)
NI_1ASY_	16	0.94 (0.003)	14	1.00 (0.00)	16	1.00 (0.00)	31	0.87 (0.004)	20	1.00 (0.00)	19	1.00 (0.00)
NI_2_	15	0.20 (0.01)	23	0.52 (0.01)	16	0.25 (0.01)	26	0.15 (0.01)	17	0.59 (0.01)	14	0.36 (0.02)
CS_1SY_	4	6.26 (0.75)	5	6.00 (0.84)	5	6.40 (0.51)	3	4.67 (0.66)	6	5.06 (0.79)	6	7.17 (0.40)
CS_1ASY_	7	6.33 (0.31)	8	7.00 (0.42)	8	7.13 (0.39)	20	6.05 (0.43)	15	6.23 (0.31)	15	7.07 (0.38)
CS_2SY_	0	NA	1	4.61 (0.40)	0	NA	0	NA	3	3.67 (0.40)	0	NA
CS_2ASY_	1	4.64 (0.30)	5	5.31 (0.30)	1	5.44 (0.30)	2	4.36 (0.30)	7	4.54 (0.30)	2	5.38 (0.30)
*H*	59	0.95 (0.001)	59	0.95 (0.001)	59	0.95 (0.001)	59	0.95 (0.001)	59	0.95 (0.001)	59	0.95 (0.001)
NS_1SY_	6	0.55 (0.12)	8	0.49 (0.14)	9	0.57 (0.12)	3	0.50 (0.15)	8	0.53 (0.10)	5	0.58 (0.14)
NS_1ASY_	15	0.19 (0.12)	12	0.23 (0.11)	15	0.37 (0.12)	27	0.29 (0.07)	19	0.42 (0.13)	18	0.36 (0.10)
NS_2_	3	0.17 (0.21)	7	0.65 (0.19)	4	0.43 (0.25)	4	0.23 (0.21)	11	0.65 (0.17)	3	0.20 (0.21)
*S*_chick_	5	0.21 (0.08)	9	0.24 (0.10)	12	0.38 (0.06)	8	0.33 (0.08)	19	0.25 (0.06)	10	0.18 (0.05)
*S*_juv_	30	0.34 (0.07)	14	0.56 (0.07)	17	0.94 (0.07)	35	0.76 (0.07)	27	0.73 (0.07)	23	0.68 (0.07)
*S*_breedSY_	16	0.47 (0.09)	8	0.64 (0.09)	13	0.83 (0.06)	13	0.75 (0.07)	17	0.73 (0.07)	6	0.70 (0.06)
*S*_breedASY_	30	0.55 (0.07)	14	0.70 (0.08)	17	0.87 (0.05)	35	0.77 (0.05)	27	0.77 (0.05)	23	0.76 (0.06)
*S*_fallSY_	16	0.48 (0.09)	8	0.64 (0.09)	13	0.84 (0.06)	13	0.75 (0.06)	17	0.73 (0.07)	6	0.71 (0.08)
*S*_fallASY_	30	0.55 (0.07)	14	0.70 (0.08)	17	0.87 (0.05)	35	0.80 (0.05)	27	0.78 (0.05)	23	0.76 (0.06)
*S*_winterSY_	16	0.67 (0.09)	8	0.79 (0.08)	13	0.91 (0.04)	13	0.86 (0.05)	17	0.84 (0.05)	6	0.83 (0.07)
*S*_winterASY_	30	0.72 (0.07)	14	0.83 (0.06)	17	0.93 (0.03)	35	0.89 (0.04)	27	0.87 (0.04)	23	0.86 (0.05)
*S*_SY_	16	0.20 (0.07)	8	0.37 (0.11)	13	0.68 (0.11)	13	0.53 (0.10)	17	0.50 (0.10)	6	0.46 (0.10)
*S*_ASY_	30	0.27 (0.07)	14	0.45 (0.11)	17	0.75 (0.09)	35	0.61 (0.08)	27	0.58 (0.08)	23	0.54 (0.12)

Abbreviations: 1, first attempt; 2, re‐nest; ASY, adults; CS, clutch size; *H*, egg hatch success rate; NI, nest initiation rate; NS, 27‐day nest survival; Schick, 54‐day chick survival; Sjuv, juvenile survival; SY, yearlings.

The intercept‐only model was the top ranked NS model for first nests of both age classes (Table 2). This model received 8.3 (yearlings) and 1.2 (adults) times more support than the second ranked model where NS varied by year for both yearling and adult females (Table 2). For re‐nests of both age classes combined, the model that included variation by year and a decreasing linear time trend within the 27‐day nesting period was the top ranked model and was 1.6 times more likely than the second ranked, intercept‐only model (Table 2). Across all years, mean NS was higher for the first nests of yearlings (NS_1SY_ = 0.54, 95% CI: 0.51–0.57) compared to the first nests of adults (NS_1ASY_ = 0.31, 95% CI: 0.24–0.38) and re‐nests (NS_2_ = 0.39, 95% CI: 0.21–0.57, Table 1).

**TABLE 2:** Model selection results for nest survival models which assessed the influence of temporal trends on 27‐day nest survival of female greater sage‐grouse (*n* = 177 nests) in the Trout Creek Mountains, Harney and Malheur counties, Oregon, USA, 2013–2018

Model	*K*	∆AICc	*wᵢ*	Deviance
*SY first nests*				
Intercept	1	0.00	0.83	161.95
Year	6	4.30	0.10	156.14
Year + Julian date	7	6.26	0.04	156.07
Year + T	7	6.33	0.04	156.14
Day	27	22.03	0.00	129.86
Year + day	32	27.18	0.00	124.15
Year × day	162	334.48	0.00	83.06
*ASY first nests*				
Intercept	1	0.00	0.42	564.66
Year	6	0.52	0.32	555.13
Year + Julian date	7	2.27	0.13	554.86
Year + T	7	2.40	0.13	554.99
Day	27	15.23	0.00	526.94
Year + day	32	16.39	0.00	517.73
Year × day	162	210.66	0.00	417.17
*SY + ASY re‐nests*				
Year + T	7	0.00	0.40	134.33
Intercept	1	0.93	0.25	147.44
Year	6	0.94	0.25	137.31
Year + Julian date	7	2.82	0.10	137.15
Year + day	32	22.57	0.00	103.35
Day	27	23.99	0.00	115.85
Year × day	162	357.71	0.00	60.82

Note

Models were ranked according to Akaike's Information Criterion with a bias correction term for small sample size (AICc) and we report ∆AICc, Akaike weight (*wᵢ*), number of parameters (*K*), and model deviance for all models.

Abbreviations: ASY, adults; SY, yearlings; T, linear trend.

Detection (*ρ*) for chicks during brood‐rearing was best described by an increasing linear time trend (i.e., detection increased with brood age, *ρ* range: 0.29–0.93, Table 3). The best model structure for chick survival included annual variation and an increasing linear time trend on count interval survival within the brood‐rearing period (Table 3). This model received 1.7 times more support than the only other competitive model where chick survival varied between years (Table 3). Chick survival to 54‐day post‐hatch was highest during 2015 and 2016 and averaged 0.27 (95% CI: 0.20–0.33) over the 6‐year study period (Table 1).

**TABLE 3:** Model selection results for chick survival models which assessed the influence of temporal trends on 54‐day chick survival of female greater sage‐grouse (*n* = 63 broods) in the Trout Creek Mountains, Harney and Malheur counties, Oregon, USA, 2013–2018

Model	*K*	∆AICc	*wᵢ*	Deviance
φ(year + T) p(T)	9	0.00	0.48	437.75
φ (year) p(T)	8	1.00	0.29	440.99
φ (year + day) p(T)	12	2.42	0.14	433.27
φ (year) p(day)	11	3.92	0.07	437.10
φ (year) p(year +T)	13	6.69	0.02	435.18
φ (year × T) p(T)	14	10.30	0.00	436.40
φ (year) p(year + day)	16	11.19	0.00	432.41
φ (int) p(int)	2	75.36	0.00	528.20
φ (year) p(int)	7	81.01	0.00	523.21

Note

Models were ranked according to Akaike's Information Criterion with a bias correction term for small sample size (AICc) and we report ∆AICc, Akaike weight (*wᵢ*), number of parameters (*K*), and model deviance for all models.

Abbreviation: T, linear trend.

Survival of female sage‐grouse varied by year, with an additive effect of age (adult > yearling) and higher survival observed during the winter months (December–March) compared to other seasons (Table 4). This top ranked model was 2.7 times more likely than the second ranked model in which survival varied across all 3 seasons (Table 4). Annual survival across all years was 0.43 (95% CI: 0.30–0.56) for yearlings and 0.51 (95% CI: 0.38–0.64) for adults. Survival increased each year after the fire until 2015, then declined slightly each year from 2016 to 2018 (Table 1). Because juvenile survival was derived from these estimates, annual variation was identical (6‐year avg. *S*_juv_ = 0.64 95% CI: 0.48–0.80).

**TABLE 4:** Model selection results for monthly survival models which assessed the influence of temporal trends on monthly survival of female greater sage‐grouse (*n* = 165) in the Trout Creek Mountains, Harney and Malheur counties, Oregon, USA, 2013–2018

Model	*K*	∆AICc	*wᵢ*	Deviance
Year + age + winter (Dec–Mar)	8	0.00	0.61	150.33
Year + age + season	9	2.00	0.23	150.31
Year + age	7	5.49	0.04	157.85
Year + age + breeding (Apr–Jul)	8	5.52	0.04	155.86
Year + age + month + winter (Dec–Mar)	15	5.53	0.04	141.63
Year + age + fall (Aug–Nov)	8	6.59	0.02	156.93
Year + age + month + breeding (Apr–Jul)	15	7.61	0.01	143.71
Year + age + month	18	9.43	0.01	139.40
Year × age	12	11.06	0.00	153.28
Intercept	1	13.81	0.00	178.24
Year × month + age	73	43.78	0.00	56.59
Year × age × month	144	152.57	0.00	0.00

Note

Models were ranked according to Akaike's Information Criterion with a bias correction term for small sample size (AICc) and we report ∆AICc, Akaike weight (*wᵢ*), number of parameters (*K*), and model deviance for all models.

### 3.2 Finite rate of population change

**FIGURE 1:**
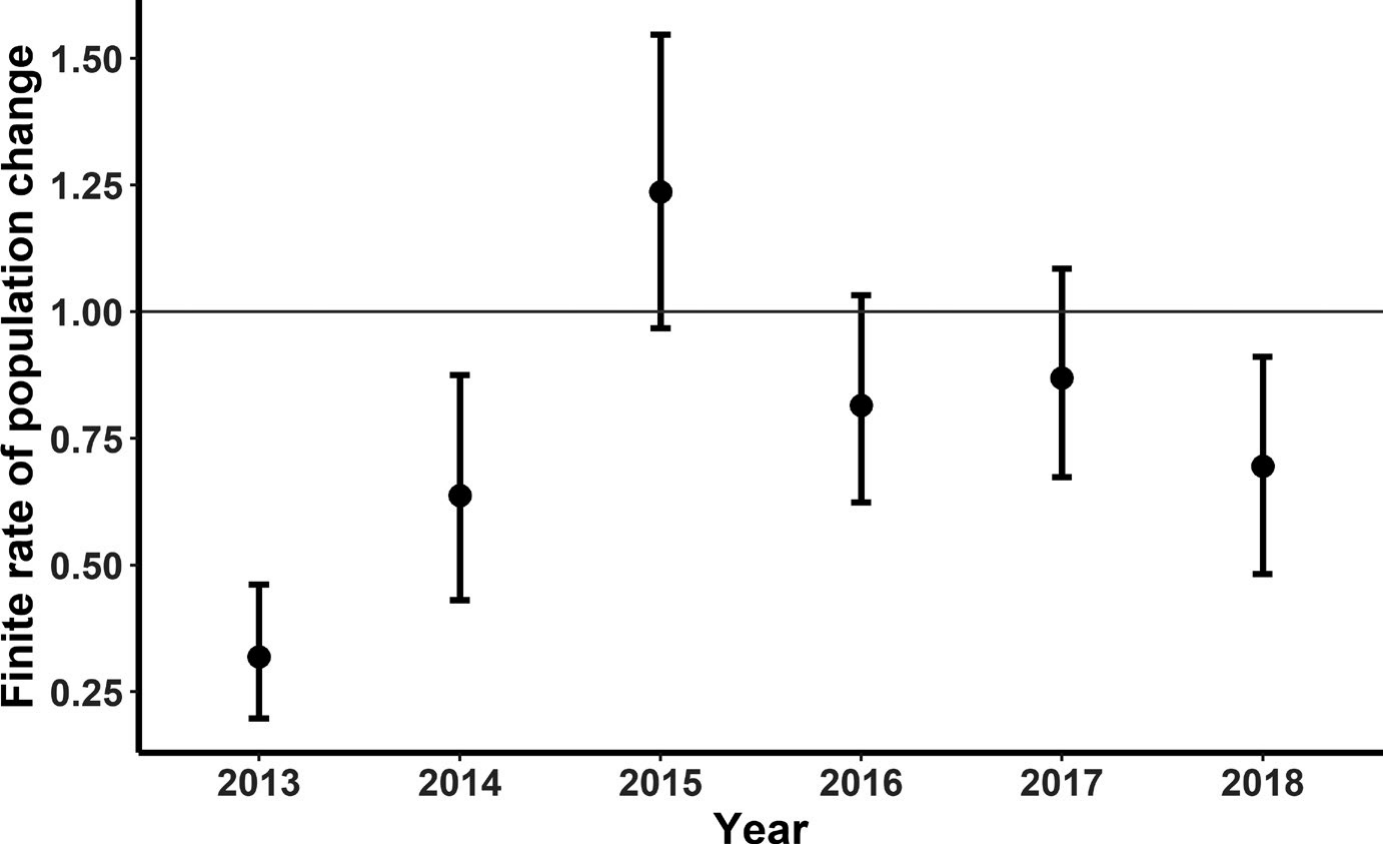
Mean finite rate of population change (*λ*) and 95% confidence intervals of female greater sage‐grouse in the Trout Creek Mountains, Harney and Malheur counties, Oregon, USA, 2013–2018. Line at 1.00 represents a stable population

Annual point estimates of *λ* indicated that the sage‐grouse population declined (*λ* ≤ 1.0) during all years except 2015. Notably, during the first 2 years after the fire, *λ* indicated declines, but *λ* increased from 2013 to 2015 (Figure 1), at which time the *λ* point estimate exceeded 1.0. However, from 2016 to 2018, the population declined again (Figure 1), with estimates of *λ* falling below 1.0 after 2015. From 2015 to 2017, there was some uncertainty around whether the sage‐grouse population was increasing, stable, or declining as 95% confidence limits overlapped 1.0 for all estimates in those years, but population declines were supported in 2013, 2014, and 2018. The geometric mean *λ* over all 6 years was 0.63 (95% CI: 0.20–1.06).

### 3.3 Life table response experiment

**FIGURE 2:**
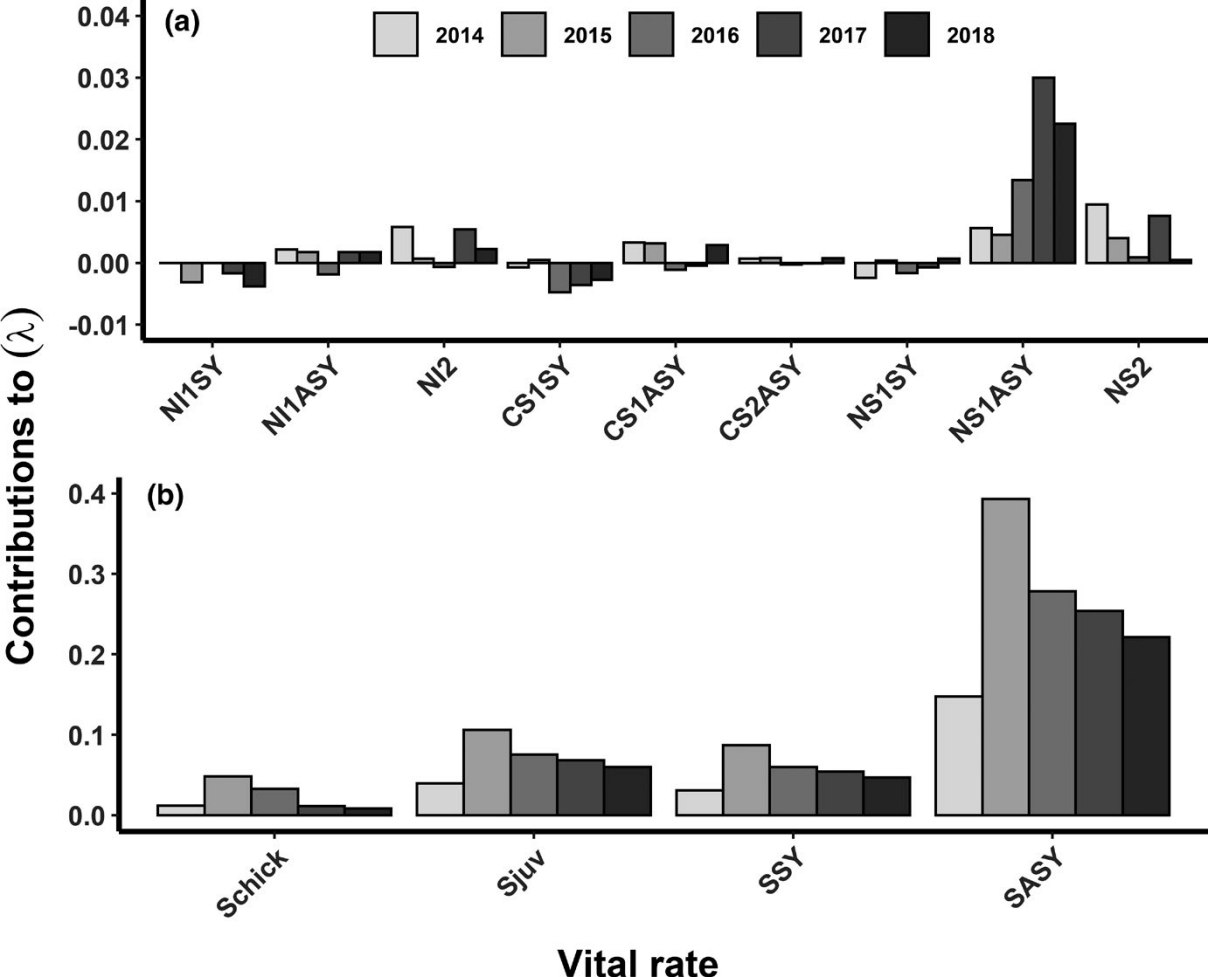
Contributions of fecundity vital rates (a) and fecundity and survival vital rates (b) to variation in population growth (*λ*) from life table response experiments for female greater sage‐grouse in the Trout Creek Mountains, Harney and Malheur counties, Oregon, USA, 2013–2018. 1, first nest; 2, re‐nest; CS, clutch size; NI, nest initiation; NS, nest survival; SASY, adult survival; Schick, chick survival; Sjuv, juvenile survival; SSY, yearling survival. Graphs depict different scales

With regards to fecundity, changes in adult NS for first nests and chick survival made the greatest contributions to variation in *λ*, and both vital rates had a positive effect on *λ* (Figure 2). Adult NS for first nests generally had an increasing positive effect and chick survival had a decreasing positive effect on *λ* with time‐since‐fire (Figure 2). The total contributions of adult NS for first nests (0.12) and chick survival (0.11) to changes in *λ* was higher than the remaining fecundity vital rates combined (0.04) for the study period. However, fecundity had less of an effect on *λ* when compared to female survival (Figure 2). Adult survival made the greatest positive contributions to changes in *λ* during each year, followed by juvenile survival and yearling survival. All three survival rates made greater contributions to *λ* than all of the fecundity rates combined during the study period and generally had a decreasing positive effect on *λ* with time‐since‐fire (Figure 2; SASY = 1.29, *S*juv = 0.35, SSY = 0.328).

## 4 DISCUSSION

Within the Great Basin, mega‐wildfires have occurred more frequently during the last 35 years, largely due to the positive feed‐back relationship between invasive annual grasses and fire (Brooks et al., 2015). Habitat loss and fragmentation due to wildfire are primary threats to sage‐grouse in the western portion of their distribution (Coates et al., 2015, 2016). Our examination of the demographic and population response of a sage‐grouse population to a mega‐wildfire suggested both acute and potentially longer‐term negative effects on sage‐grouse populations. We observed strong evidence of an annual population decline during 3 of 6 years following the fire, with more uncertainty observed in 2015 when populations may have been increasing, and 2017 and 2018, when populations may have been declining. These dynamics were driven largely by female survival and to a lesser extent nest and chick survival. Approximately, 75% of the study area burned during the Holloway fire (Foster et al., 2018) and <5% shrub cover remained in burned areas (compared to 20% in unburned areas, Anthony et al., 2020). We suspect that carrying capacity decreased after the fire due to the vast amount of sagebrush loss and philopatric behavior of the species thus, patterns in *λ* during the first 3 years post‐fire were consistent with what we would predict under an extreme disturbance. The continued decline in *λ* since 2016 suggested the loss of 186,972 ha of sagebrush habitat and slow regeneration of sagebrush plants post‐fire may prolong recovery of sage‐grouse after a mega‐wildfire (Steenvoorden et al., 2019). Mean estimates of *λ* during 2016–2018 were much lower in the Trout Creek study area (0.75) compared to a study in Oregon during the same time period that was not affected by fire (0.93; Olsen et al., 2021). Moreover, estimates of *λ* were low compared to other studies that reported long‐term trends in *λ* across the distribution of sage‐grouse (Dahlgren et al., 2016; Garton et al., 2011; Johnson & Braun, 1999).

There was temporal variation in annual vital rate contributions to *λ* following the fire. Overall, the contribution of female survival declined while the contribution of NS increased with time since fire, suggesting transient dynamics of a population post disturbance (Koons et al., 2017). Female survival had an increasing, positive effect on *λ* during the first 2 years after the fire, then the strength of contributions declined annually thereafter. Whereas, NS had an increasing, positive effect during the first 5 years and then declined slightly during the final year. Unlike most galliformes where fecundity is the primary driver of population growth, sage‐grouse demonstrate greater variation in demographic tactics across their range (Taylor et al., 2012). However, the negative effects of the fire appear to outweigh the benefits of this variation as given by a declining population in 3 of 6 years post‐fire.

Adult survival was a key driver of population growth for sage‐grouse after the Holloway fire. Estimates of adult survival were low relative to values reported from across the species range (Taylor et al., 2012). Temporal patterns in annual female survival showed that survival was lowest during the first 2 years post‐fire ($\bar{x}$ = 0.36, SD = 0.13), then survival improved to its highest rate during 2015, with declines observed in each of the last three years ($\bar{x}$ = 0.58, SD = 0.04) to estimates that are below species norms in undisturbed systems (i.e., 0.62; Taylor et al., 2012). Interestingly, while our estimates of female survival were lower overall, the annual patterns we observed were similar to those observed for females in a concurrent Oregon study without wildfire effects (2013–2018; Olsen et al., 2021). Seasonal variation within years suggested that winter survival was highest, as expected for the species (Blomberg, Sedinger, et al., 2013; Moynahan et al., 2006; Walker, 2008). Adults in our study experienced mortality peaks in April and October. Mortalities during October coincide with increased raptor abundance during fall migration and are commensurate with sage‐grouse transitioning from summer to winter ranges (Foster, 2016). We speculate that the lack of sagebrush cover may have increased mortality risk as birds transition between seasonal habitats. The month of April was the peak of nest initiation and incubation; however, only 1 of our marked females was killed while incubating. Thus, though they were not depredated while incubating, they may have been more susceptible to predation given the lack of sagebrush to conceal them as they moved from their nest site to foraging locations during incubation recesses.

NS for adults was substantially lower during the first 2 years ($\bar{x}$ = 0.21, SD = 0.03), but then increased during years 3 through 6 post‐fire ($\bar{x}$ = 0.36, SD = 0.07) when we observed estimates that were low but more comparable to those reported across the species range ($\bar{x}$ = 0.44; Taylor et al., 2012). Weather conditions and snowpack restrict nesting activities in the study area to Wyoming big sagebrush which has low resistance to fire and resiliency to invasive annual grasses. The lack of sagebrush cover ($\bar{x}$ = 2%) and high cheatgrass cover ($\bar{x}$ = 12%) provided low quality nesting habitat within burned Wyoming big sagebrush communities in our study area (Anthony et al., 2020). Despite these unfavorable nesting conditions, sage‐grouse selected fine‐scale vegetation features within burned areas, which provided thermal and concealment cover, a strategy which increased NS (Anthony et al., 2021).

The chick survival estimates we observed were contrary to our expectations that the increase in nutrient‐rich forbs post‐fire in mountain big sagebrush communities, where late brood‐rearing occurs, would be beneficial to chick survival. Low estimates suggests that other environmental conditions in Wyoming big sagebrush communities during early brood‐rearing (e.g., changes in plant species composition, lack of screening and thermal cover, changes in predator community) could be limiting chick survival post mega‐wildfire (Anthony et al., 2020; Fischer et al., 1996). Model‐averaged estimates of chick survival (6 year, avg = 0.27, SD = 0.08) were some of the lowest estimates reported for sage‐grouse (Taylor et al., 2012). Of the studies that estimated chick survival over a similar time period as our study, only 2 have reported lower average estimates (56 day: 0.12, 54 day: 0.20; Aldridge & Boyce, 2007; Schreiber et al., 2016) and in these cases oil well densities and drought conditions were limiting chick survival. However, estimates of chick survival exhibited a similar trend to those reported from another study in Oregon from 2013 to 2017, indicating regional‐scale factors might have contributed to decreases observed in our study (e.g., weather, Olsen et al., 2021). Variation in chick survival has been linked to numerous environmental factors including food resources, vegetation cover, nest locations, drought, temperature, precipitation, and predators (Gibson et al., 2016, 2017; Gregg & Crawford, 2009; Guttery et al., 2013; Schreiber et al., 2016). Chick survival did increase during the brood‐rearing period as expected in conjunction with growth factors in chicks such as thermoregulatory effectiveness, foraging efficiency, and predator avoidance (Nichelmann & Tzschentke, 2002), and higher quality brood‐rearing habitat. Regardless, estimates were lower than expected as we anticipated that the abundance of food resources available after the fire would have bolstered chick survival. We also acknowledge that our estimates of chick survival may be inflated because we were not able to account for potential brood adoption because there are no published methods to correct for the effects of adoption rates on estimates of chick survival. However, there are numerous published studies on sage‐grouse and other galliformes using this same methodology (Gibson et al., 2016; Hagen et al., 2009; McNew et al., 2012; Olsen et al., 2021).

Predation is the primary cause of mortality in sage‐grouse across their range (Blomberg, Gibson, et al., 2013; Connelly, Hagen, et al., 2011; Hagen, 2011) and increased rates of predation could limit female and chick survival following wildfires that remove a large portion of sagebrush cover (Dinkins et al., 2014). Sage‐grouse have high fidelity to their seasonal ranges, despite extreme reductions in vegetation cover and concealment of birds and nests in the post‐burned landscape (Foster et al., 2019). In addition, most species that commonly prey on sage‐grouse presumably did not disperse following fire because they use a wide range of vegetation types (Vander Haegen et al., 2001). Within the Holloway fire perimeter, only 25% of the landscape was unburned, leaving substantially less cover for sage‐grouse post‐fire (Foster et al., 2019). Annual and perennial grasses have reoccupied the burned areas, and some mammalian predators might avoid areas with high cheatgrass canopy cover (Holbrook et al., 2016). However, sagebrush is slow to recover post fire (15–100 years depending on species), and the low vegetation structure following the fire may increase predators’ ability to detect sage‐grouse, especially where topographic ruggedness is high (Dinkins et al., 2014). Thus, post‐fire restoration efforts may want to target increasing the number and size of available sagebrush patches to increase sage‐grouse survival (Steenvoorden et al., 2019).

It is important to recognize that much of our study area would be categorized as vegetation communities that have high resistance and resilience to invasive plants and large‐scale disturbance (Chambers et al., 2017, 2019). Despite the apparent slow‐recovery of sage‐grouse population growth in the Holloway Fire, recovery may be further hindered in landscapes with lower resistance and resiliency. Interactions between local weather and post‐fire vegetation may limit the amount of productive habitat for females (Donnelly et al., 2018). Additionally, drought conditions can reduce the amount of usable space for females during the nesting season and negatively impact chick survival (Blomberg et al., 2017; Gibson et al., 2017). Mean annual temperature was 1.6°C warmer and annual precipitation accumulation was 26.5 mm lower during the study period when compared to 30‐year averages. We observed the lack of vegetation community recovery in localized sites within our study area, and the absence of sage‐grouse used therein (Schuyler, E. M., Hagen, C. A., Anthony, C. R., Foster, L. J., Dugger, K. M., Unpublished data). We surmise that extended periods of recovery or permanent losses to irreversible state transitions of invasive annual grasses will continue to limit recovery of sage‐grouse in the fire perimeter or other communities of low resistance and resilience (Chambers et al., 2017; Coates et al., 2016). Our work contributes to the growing body of evidence on acute and chronic effects of fire on sage‐grouse (Coates et al., 2016; Dudley et al., 2021) and suggests that prevention of fire may be the most effective strategy in retaining large landscapes of sagebrush. In the near term, proactive approaches to reduce wildfire risk in low resistance sagebrush communities may be a reasonable strategy to minimize long‐term effects of wildfire on sage‐grouse populations. Additionally, targeted approaches to expedite recovery of sagebrush communities may buffer lag effects of wildfire on sage‐grouse populations.

## CONFLICT OF INTERESTS

All contributing authors have approved this manuscript and have agreed to Ecology and Evolution's submission policies. The authors declare that they have no known competing financial interests or personal relationships that could have appeared to influence the work reported in this paper. This manuscript has not been previously published and is not currently in review by another journal. Capture and handling of all greater sage‐grouse were conducted under protocols approved by the Institutional Animal Care and Use Committee at Oregon State University.

## AUTHOR CONTRIBUTIONS

**Christopher R. Anthony:** Data curation (lead); Formal analysis (lead); Investigation (equal); Validation (lead); Writing – original draft (lead). **Lee J. Foster:** Data curation (equal); Investigation (equal); Writing – review & editing (equal). **Christian A. Hagen:** Conceptualization (equal); Funding acquisition (equal); Methodology (equal); Project administration (equal); Resources (equal); Supervision (equal); Writing – review & editing (equal). **Katie M. Dugger:** Conceptualization (equal); Funding acquisition (equal); Methodology (equal); Project administration (equal); Resources (equal); Supervision (equal); Writing – review & editing (equal).

## Data Availability

Data R script are available as a Dryad repository, https://doi.org/10.5061/dryad.08kprr53z.
